# Band Gap Tunable Zn_2_SnO_4 _Nanocubes through Thermal Effect and Their Outstanding Ultraviolet Light Photoresponse

**DOI:** 10.1038/srep06847

**Published:** 2014-10-30

**Authors:** Yan Zhao, Linfeng Hu, Hui Liu, Meiyong Liao, Xiaosheng Fang, Limin Wu

**Affiliations:** 1 Department of Materials Science, Fudan University, Shanghai 200433, P. R. China; 2 Optical and Electronic Materials Unit, National Institute for Materials Science (NIMS), Namiki 1-1, Tsukuba, Ibaraki 305-0044, Japan

## Abstract

This work presents a method for synthesis of high-yield, uniform and band gap tunable Zn_2_SnO_4_ nanocubes. These nanocubes can be further self-assembled into a series of novel nanofilms with tunable optical band gaps from 3.54 to 3.18 eV by simply increasing the heat treatment temperature. The Zn_2_SnO_4_ nanocube-nanofilm based device has been successfully fabricated and presents obviously higher photocurrent, larger photocurrent to dark current ratio than the previously reported individual nanostructure-based UV-light photodetectors, and could be used in high performance photodetectors, solar cells, and electrode materials for Li-ion battery.

Zinc stannate (Zn_2_SnO_4_) is one of the most important ternary metal oxides and has attracted considerable attention because of its unique optical, electrochemical and photoelectrochemical properties. For example, previous researches have disclosed that Zn_2_SnO_4_ could be used as working electrodes in dye-sensitized solar cells due to its high electron mobility and fast electron transport^1^, and as anodes for Li-ion battery due to its high theoretical capacity and reversible capacity^2^,^3^,^4^. Moreover, Zn_2_SnO_4_ also exhibits high gas sensitivity and rapid response as gas sensors^5^,^6^, and photocatalytic degradation of organic pollutants in aqueous solutions due to its high charge separation induced by surface oxygen-vacancies states^7^,^8^.

On the other hand, photodetection in UV region has drawn considerable attention due to its extensive applications including environmental and biological fields, optical communications, sensors, and missile-launch detection^9^,^10^,^11^. So far, a variety of thin-film based photodetectors such as GaN^12^, ZnS^13^, ZnO^14^,^15^,^16^ and TiO_2_^17^, have been fabricated and investigated for UV irradiation detection, due to their wide band gaps and fast response speeds. However, there still exist some drawbacks in the fabrication of thin-film based photodetectors. For example, the commercial fabrication method of GaN thin-film photodetector using metal organic chemical vapor deposition method is troublesome and costly^12^. ZnO thin-films are usually fabricated by complicated and expensive vacuum deposition system or a time consuming sol-gel process^5^,^16^. Therefore, it is of great importance to develop new materials and facile fabrication processes for high-performance photodetectors.

Recently, we have reported a series of nanostructure-based nanofilm photodetectors by an oil-water interfacial self-assembly strategy^18^,^19^,^20^. This novel strategy effectively opens the door for the self-assembly of hydrophilic nanostructures into closely-packed nanofilms, and provides a facile method to construct thin-film based nanodevices. Compared to the previously reported strategies such as spin coating, vertical deposition or dip coating, oil-water interfacial self-assembly method can be used to fabricate monolayer films, and the periodic structures of nanofilms are much better controlled^21^,^22^,^23^,^24^,^25^,^26^,^27^. Yet there are still no reports on the UV photodetectors using Zn_2_SnO_4_ nanocubes as the building blocks and their optoelectronic properties have been rarely investigated to the best of our knowledge, although Zn_2_SnO_4_ is a very promising candidate for UV light detection because of its proper band gap (*E*_g_) around 3.7 eV^28^. On the other hand, photodetectors with tunable band gaps are very attractive due to their applications for various regions of the spectrum^29^,^30^. In general, band gaps of the semiconducting nanocrystals may be controlled by the size or composition of the nanocrystals, such as HgTe nanocrystals, (Cu_2_Sn)*_x/3_*Zn*_1−x_*S nanoparticles and Cu_2_ZnSn(S*_1−x_*Se*_x_*)_4_ nanocrystals^31^,^32^,^33^. In this study, we present a chemical method for synthesis of high-yield, uniform and band gap tunable Zn_2_SnO_4_ nanocubes and further fabricate a high-performance UV light photodetector by employing Zn_2_SnO_4_ nanocubes as shown in Fig. 1. Interestingly, the optical band gaps of the Zn_2_SnO_4_ nanocube-based films can be tuned from 3.18 to 3.54 eV through a heat treatment process, and the optimal band gap of Zn_2_SnO_4_ nanofilm is especially suitable for UV-A (320–400 nm) light detection. This Zn_2_SnO_4_ nanocube-based device displays high photocurrent, large photocurrent to dark current ratio, excellent stability, and reproducibility, which are considerably better than the previously reported values.

## Results

**Figure 1:**
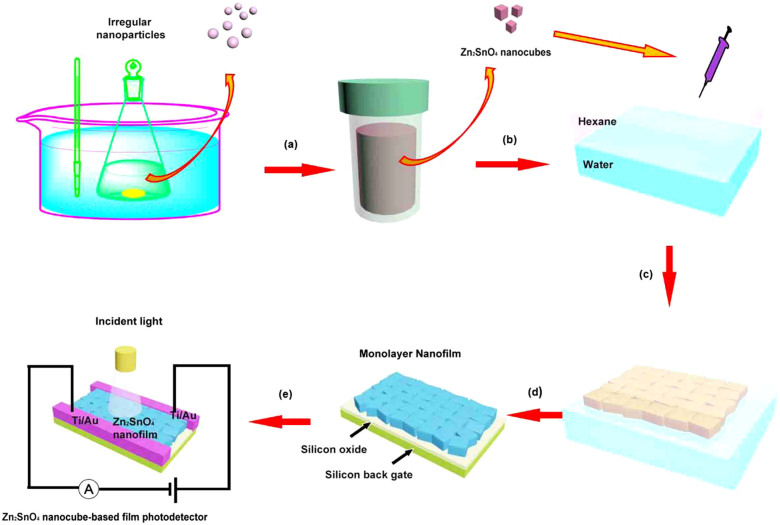
Schematic illustration of the fabrication procedure of the Zn_2_SnO_4_ nanofilm and photoresponse nanodevices. (a) Water bath and hydrothermal process. (b)–(c) Hexane-water interfacial self-assembly. (d) Lift-up process and heat treatment process. (e) Schematic illustration of photoresponse nanodevice.

**Figure 2:**
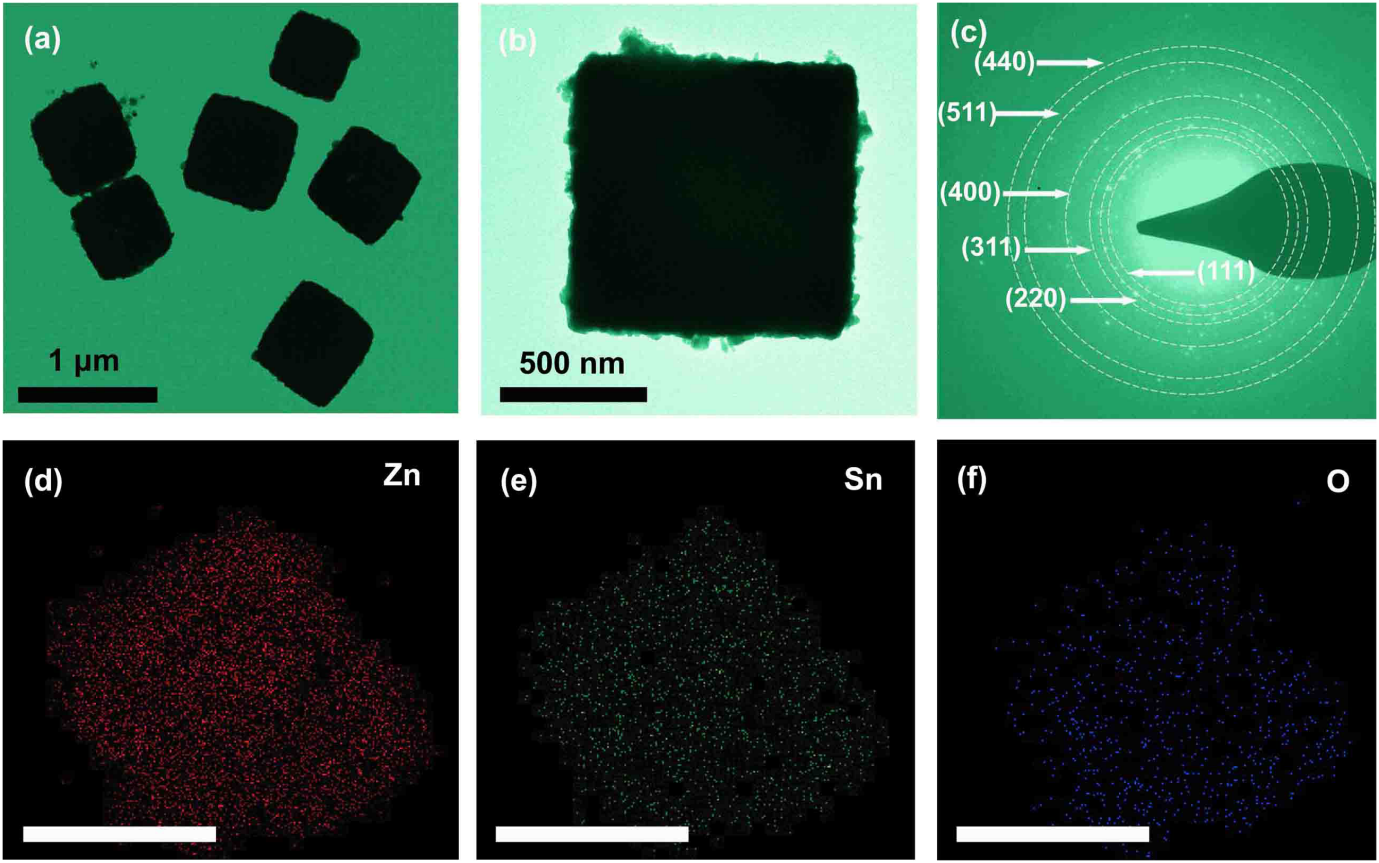
(a) and (b) TEM images of Zn_2_SnO_4_ nanocubes with different magnifications. (c) Corresponding SAED pattern taken from a single Zn_2_SnO_4_ nanocube. (d), (e) and (f) Zn, Sn and O elemental maps, respectively, scale bar: 500 nm.

Zn_2_SnO_4_ nanocubes were fabricated by a water bath and a hydrothermal method as shown in Fig. 1a. First, Zn(CH_3_COO)_2_·2H_2_O, SnCl_4_·5H_2_O and sodium dodecyl benzene sulfonate (SDBS) were added into a mixed solution of ethanol and distilled water in a conical flask which was then put in a water bath under magnetic stirring at 60°C. Subsequently, tetraethylammonium hydroxide (TEAH) was added dropwise to the stirred solution as the structure-directing agent. After continuously stirred for 1 h, the suspension was then transferred into a 50 mL Teflon-lined stainless steel autoclave. Finally, Zn_2_SnO_4_ nanocubes were fabricated by a hydrothermal method and a subsequent annealing procedure, with a high yield of 62%. Fig. 2a and b show the typical transmission electron microscopy (TEM) images of the as-prepared product synthesized in the hydrothermal system at 220°C for 5 h and heated at 500°C for 1 h. It is confirmed that the cube morphology was well maintained during the annealing treatment. Fig. 2c shows the SAED pattern of a single Zn_2_SnO_4_ nanocube, which proves to be a polycrystalline structure in nature. In order to further confirm the chemical composition and elemental distribution, scanning transmission electron microscope (STEM) studies were performed. As displayed in Fig. 2d–f, the Zn, Sn and O elements are homogeneously distributed in this nanocube. The final product has surface area of 10.92 m^2^ g^−1^ and pore volume of 0.083 m^3^ g^−1^ based on Brunauer–Emmett–Teller (BET) and Barrett–Joyner–Halenda (BJH) in Supplementary Fig S1.

**Figure 3:**
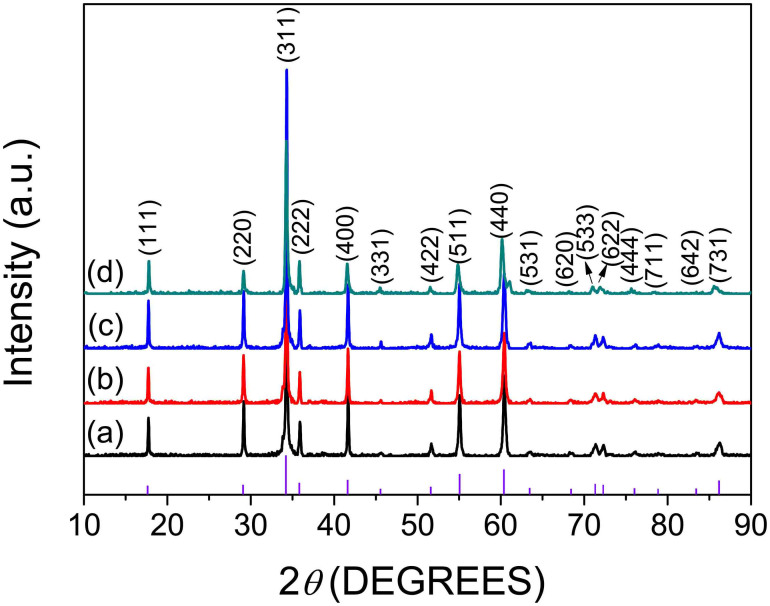
XRD patterns of Zn_2_SnO_4_ nanocubes with different heating temperature. 60°C (a), 200°C (b), 300°C (c) and 500°C (d) for 1 h. JCPDS 24–1470 pattern is shown for comparison (vertical lines).

According to the XRD patterns of Zn_2_SnO_4_ powder samples in Fig. 3, all the diffraction peaks are in good agreement with the data of pure cubic inverse spinel phase of Zn_2_SnO_4_ (JCPDS: 24–1470) and no extra peak is detected, indicating that all the as-prepared samples are of high purity phase and in perfect crystallinity. Based on the XRD peaks of Zn_2_SnO_4_ and the Scherrer formula (*ϕ* = *kλ*/*β*cos*θ*) applied to the prominent peaks corresponding to the plane (311), the lattice constant of Zn_2_SnO_4_ samples with different heating temperature were found to be 8.656, 8.658, 8.659 and 8.689 Å, respectively. Despite small differences of lattice constant that exist among the samples, all of them shared the same trends of variation of the crystal parameters, which corroborated the homogenous nature of the nanocrystals.

Reaction temperature in hydrothermal condition showed an obvious influence on the formation of the Zn_2_SnO_4_ nanocubes. Schematic illustration for the possible formation mechanism of the as-prepared samples can be described in Supplementary Fig S2. Firstly, a series of irregular ZnSn(OH)_6_ and ZnO nanoparticles were formed in the mixture with the assistance of SDBS before a hydrothermal reaction. The pH of the starting solution was 13.3. Then, ZnSn(OH)_6_ nanocubes with a little ZnO nanoparticles on the surface were obtained and Zn_2_SnO_4_ nanocubes started to generate when the temperature rose to 180°C. The pH of system at this time was decreased to 11. From the XRD pattern (Supplementary Fig S3a), we can see that ZnSn(OH)_6_ nanocubes were formed at 180°C. The peaks of as-prepared product match well with the cubic phase of ZnSn(OH)_6_ (JCPDS: 33–1376). Only three small diffraction peaks of hexagonal ZnO are detected, which are caused by the decomposition of 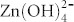. With the increase of the hydrothermal reaction temperature, more Zn_2_SnO_4_ nanocubes have been formed. ZnSn(OH)_6_ nanocubes can be used as a reactants and a self-sacrifice template to form uniform Zn_2_SnO_4_ nanocubes (Supplementary Fig S3b). Finally, Zn_2_SnO_4 _nanocubes were completely obtained at 220°C for 5 h, and the pH value of the final solution was 10.3. With further prolongation of reaction time, the size of the as-prepared product increased rapidly. Uniform Zn_2_SnO_4_ nanocubes with the size of 2.5 μm were prepared at 220°C for 8 h (Supplementary Fig S4a). The reactions during the formation of Zn_2_SnO_4_ nanocubes can be shown as follows: 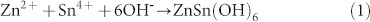

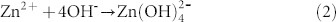

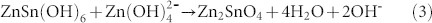

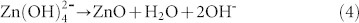


It is worth noting that the formation of Zn_2_SnO_4_ nanocubes should be strongly affected by hydroxide concentration. When the added alkaline exceeded a certain amount, the ZnO precipitates were produced as the by-product in the thermal decomposition of 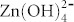. Furthermore, highly hydrated TEAH is considered as an efficient ionic liquid precursor (ILP), which cannot only act as a solvent, but also a reactant for the fabrication of inorganic materials^34^. The presence of anionic surfactant SDBS is also crucial for the formation of Zn_2_SnO_4_ nanocubes. As shown in Supplementary Fig S4b and c, Zn_2_SnO_4_ nanoplates and blocks were produced without SDBS at 200°C for 20 h and 220°C for 5 h, respectively. SDBS might guide the formation of cube ZnSn(OH)_6 _precursors, which further converted to the cubic Zn_2_SnO_4_ nanostructure. We could also conclude that the general morphology and the average size of the Zn_2_SnO_4_ nanostructure were strongly influenced by the reaction temperature, the reaction time and the composition of the reactants.

**Figure 4:**
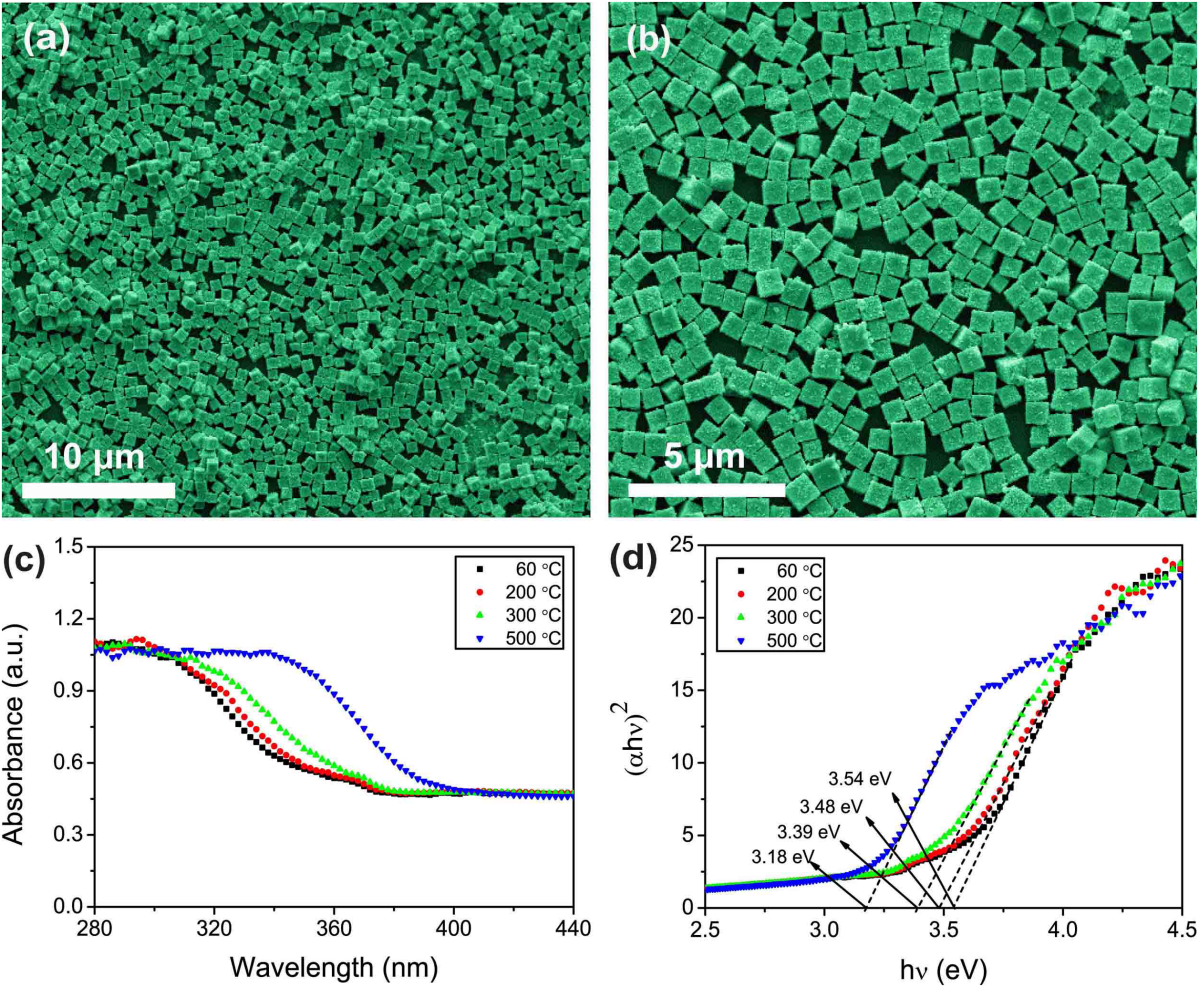
(a) Low- and (b) high-magnification SEM images of the Zn_2_SnO_4_ nanocube-based film. (c) Typical room-temperature UV-visible absorbance spectra and (d) the plot of (*αhν)^2^ vs hν* of the Zn_2_SnO_4_ nanofilm on quartz substrate with different calcined temperature.

As shown in Fig. 1b–d, Zn_2_SnO_4_ nanocube-based monolayer nanofilms were easily fabricated using an oil-water interfacial self-assembly strategy and a calcining process. The optical microscopy images of the Zn_2_SnO_4 _monolayer nanofilm deposited on silicon substrates and quartz substrates under natural light are shown in Supplementary Fig S5, respectively. It can be clearly seen that the nanofilms are uniform and semitransparent, further indicating the high quality of the nanofilms. The SEM images in Fig. 4a and b show an overview of the monolayer Zn_2_SnO_4_ nanofilms with different magnifications. It is apparent that the substrate is densely covered by a large number of regular Zn_2_SnO_4_ nanocubes with an average edge length of 650 nm.

Fig. 4c shows the UV–vis absorption spectra of the Zn_2_SnO_4_ nanofilm on quartz substrate with different annealing temperature. All of the samples display a strong absorption edge at 375, 381, 412 and 424 nm, respectively. Note that the heat-treated samples show a shift of the absorbance cutoff to higher wavelengths, which indicates a decrease in the optical *E*_g_ after different heat treatment temperature. The red-shift of the absorption edge compared to that of the nanofilm heat treatment at 60°C might be closely associated with the change of the average grain sizes and the lattice constant of the as-prepared samples^35^. As shown in Fig. 4d, the band gaps of the materials at different heat treatment temperature could be estimated to be 3.54, 3.48, 3.39 and 3.18 eV, respectively, which are apparently smaller than that before heat treatment with an apparent red-shift of about 0.30–0.50 eV. Apparently, the product after heat treatment is especially suitable for the UV light detection due to the decreased *E*_g_, especially in the UV-A area. The band gap energy of Zn_2_SnO_4_ was previously reported to vary from 3.6 eV in the bulk form to 3.43 eV in the thin film^7^,^35^. In our study, the hydrothermal method has a tendency to produce materials with a small excess of Zn despite the initial stoichiometric amount used, which has been confirmed by ICP-OES measurement as follows: the actual Zn to Sn molar ratios of three parallel sample solutions A, B and C were 2.044, 2.029 and 2.035, respectively, with a mean of 2.036. During the heat treatment procedure, the high activation energy may drive the excess of Zn infiltrating into the lattice of Zn_2_SnO_4_ and cause the defect of energy level. Alpuche-Aviles *et al.* also reported that the fundamental band-gap of Zn_2_SnO_4_ nanoparticles was 3.60–3.70 eV, and thermal treatment could narrow the band gap due to the incorporation of excess Zn into Zn_2_SnO_4_ matrix^35^. Although the optical absorption property of Zn_2_SnO_4_ is still controversial, the reported band gaps are all in the range of 3.2–3.9 eV. It is obvious that the synthetic approach and the morphology of Zn_2_SnO_4_ nanofilm have significant impacts on their optical absorption property^36^,^37^.

**Figure 5:**
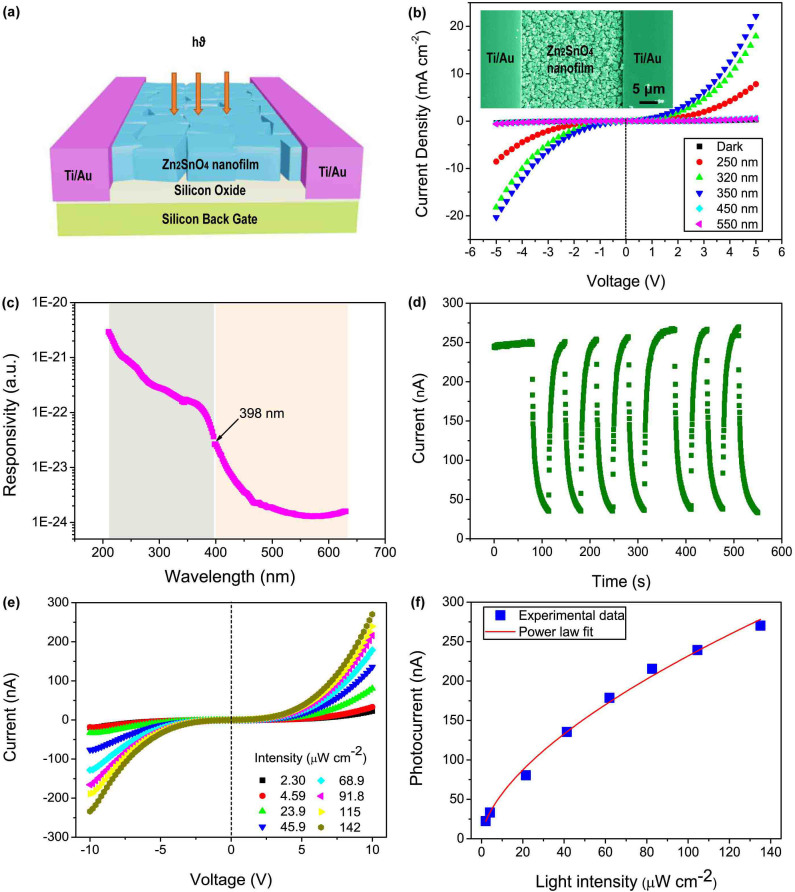
(a) Schematics of the Zn_2_SnO_4_ nanofilm photodetector. (b) The *I–V* characteristics of the device illuminated with different-wavelength lights or under dark conditions. Inset: A representative SEM image of the device. (c) A typical spectral photoresponse of the device for different wavelengths. (d) The reproducible on/off switching upon 350 nm light illumination. (e) *J–V* characteristics of the device under 350 nm light irradiation with various power intensities. (f) The light-intensity-dependent photocurrent of the device at a bias of 10 V.

The Zn_2_SnO_4 _nanocube-based nanofilms are very suitable for UV-light detection due to their optical band gaps can be tuned through thermal effect. The optimal band gap of our Zn_2_SnO_4_ nanocube, 3.18 eV, is in good agreement with the threshold wavelength of UV-A reagion, which makes it become an excellent material for UV-A sensor. For this reason, a nanocube-based nanofilm device (Fig. 1e) from the above Zn_2_SnO_4_ film after 500°C annealing was successfully constructed by a simple electron-beam deposition method similar with our previous reports^18^. A schematic diagram showing the configuration of a monolayer Zn_2_SnO_4_ nanocube-based nanofilm device for the photocurrent measurement is illustrated in Fig. 5a. The inset of Fig. 5b shows the SEM image of the device in which the monolayer nanofilm was connected by a pair of electrodes placed 30 μm apart. The *J–V* measurements of the nanofilm photodetector in the dark and under light illuminations are shown in Fig. 5b. It can be seen that the photoresponsivity just shows very slight changes when the wavelength of the light sources are 550 nm (0.252 mW/cm^2^) and 450 nm (0.322 mW/cm^2^). When the device was illuminated by a 350 nm UV light at 0.152 mW/cm^2^, a drastic increase of current density up to 22.14 mA cm^−2^ was detected at an applied voltage of 5 V (about 76 times enhancement compared with a dark current density of 0.29 mA cm^−2^). The symmetric *J−V* curves indicate good ohmic contact between the Zn_2_SnO_4_ nanocube-based thin-film and the Ti electrodes. The appearance of photoconductive sensitivity in the present Zn_2_SnO_4_-nanocube device is ascribed to the electron−hole pairs excited by the incident photons with energy larger than the band gap, that is, only the light with enough energy is able to induce a significant increase in conductance. The photocurrent of the Zn_2_SnO_4 _nanofilm device is three orders of magnitude higher than that of an individual ZnS nanobelt^38^ and 150 times enhancement compared to that of ZnO nanowire^41^ under the similar condition. The exposed area on an individual nanostructure-based nanodevice is quite limited, leading to an absolutely low photocurrent and poor repeatability^38^,^39^,^40^,^41^,^42^. Compared with the individual-nanostructure-based photodetectors, high photocurrent of the present nanofilm device might be due to a fact that the photocurrent of the device is collected from a large number of Zn_2_SnO_4_ nanocubes rather than a single one^43^. Such a drastic enhancement is very promising for practical application such as field emitters, light emission diodes (LEDs), photodiodes, etc^44^,^45^,^46^. The greatly enhanced photocurrent and photocurrent to dark current ratio suggests that the Zn_2_SnO_4_ nanocube-film-based photodetector has great advantages in improving the performance of UV-light photodetectors compared with the individual-nanostructure-based photodetectors. Furthermore, other key performance parameters of the present nanodevice are also obviously superior to those of other existing semiconducting photodetectors as summarized in Table 1.

Fig. 5c depicts the photon-response spectrum of the device as a function of the incident light wavelength from 210 to 630 nm at a bias of 10 V. We can see that the sensitivity is very low for the wavelength longer than 450 nm. This starts to gradually increase (up to one order of magnitude increase) between 398 nm (near the band gap of Zn_2_SnO_4_ (≈3.18 eV, 390 nm)) and 450 nm, and then increases two orders of magnitude when the wavelength decreases to 210 nm. The huge increases of sensitivity under UV-light illumination as compared to visible light justify that the present Zn_2_SnO_4_ nanocube-based film is indeed particularly valuable for UV-light detection.

Stability is another key parameter which determines the capability of a photodetector to follow a quickly varying optical signal. The time-dependent photoresponse of the as-constructed device is shown in Fig. 5d, which is measured by periodic turning on and off a 350-nm-light at a bias voltage of 10 V. Upon illumination, the photocurrent rapidly increases to a stable value of 257 nA on average and then decreases dramatically to its initial value (35.2 nA) when the light is turned off, giving an on/off switching ratio of 7.3. The photocurrent of the present device shows an outstanding stability and repeatability. No obvious degradation is observed after a number of cycles.

Further experiment in Fig. 5e shows that the photocurrent is very sensitive to the intensity of the incident light. The device was irradiated by a 350-nm-light at a bias of 10 V. By adjusting the intensity of illumination, the photocurrent can be reversibly changed from 22.4 nA to 270.3 nA accordingly, which may lie in the different photon densities from the incident lights. As shown in Fig. 5f, the current of the device is strongly related to the light intensity and demonstrates a power dependence of 0.61 (*C* = 14.15 × *P*^0.61^), whereas C is the photocurrent value and P is the light intensity. The non-unity exponent is a result of the complex process of electron–hole generation, trapping, and recombination within the semiconductor^47^. By simply adjusting the intensity of illumination, the current can be reversibly changed to more than one order of magnitude (about 12 times) without damaging the film.

## Discussion

In summary, the Zn_2_SnO_4_ nanocubes with well-defined morphology have been high-yieldly grown by a hydrothermal method using low-cost reagents. The optical band gaps of the Zn_2_SnO_4_ nanocubes can be easily controllable from 3.18–3.54 eV through a heat treatment process. The optimal band gap of Zn_2_SnO_4_ nanofilm is especially suitable for UV-A (320–400 nm) light detection, and the as-constructed device exhibits greatly higher photocurrent and relatively larger photocurrent to dark current ratio compared with the previously reported individual-nanostructure-based UV-light photodetectors. The high photocurrent, large photocurrent to dark current ratio, high spectral selectivity, excellent photocurrent stability and reproducibility render the present Zn_2_SnO_4_ nanocube-based nanofilm device to be particularly valuable for ultraviolet light detection, solar cells and photoelectronic switches.

## Methods

Zn_2_SnO_4_ nanocubes were synthesized by a hydrothermal method with some changes of experimental conditions^5^ and an annealing treatment at different temperature. The Zn_2_SnO_4_ nanocube-based nanofilm was fabricated using an oil–water interfacial self-assembly method (see Supporting Information for details). The current density-voltage (*J-V*) characteristics of the Zn_2_SnO_4_ nanofilm photodetector were measured using an Advantest picoammeter R8340A and a dc voltage source R6144. Spectral responses for different wavelengths were recorded by using a xenon lamp (500 W). The time-dependent photoresponses of the device were measured using a current meter after shutting off the UV light. The incident light power was calibrated using an UV enhanced Si photodiode.

## Author Contributions

Y.Z. performed the experiments. M.Y.L. provides facilities. H.L. did the device fabrication and helped on photocurrent measurement. L.M.W., L.F.H. and Y.Z. wrote the manuscript. X.S.F. and M.Y.L. discussed the content. All authors reviewed the manuscript.

## Supplementary Material

Supplementary Information Supplementary Information files

## Figures and Tables

**Table 1:** Comparison of the critical parameters for the present Zn_2_SnO_4_ nanofilm and other characteristic inorganic semiconducting nanostructure-based UV-light photodetectors

Photodetectors	Light of detection	Bias/V	Photocurrent *I*_light_	Dark current *I*_dark_	*I*_light_/*I*_dark_	References
individual ZnS nanobelt	320 nm	10	0.8 pA	0.07 pA	11.4	38
individual ZnS microbelt	320 nm	20	6.5 pA	<0.1 pA	--	39
individual ZnO nanorod	350 nm	2	22 nA	1 nA	22	40
individual ZnO nanowire	350 nm	5	280 pA	~15 pA	18.7	41
individual Nb_2_O_5_ nanobelt	320 nm	1	51.3 pA	15.5 pA	3.3	48
individual In_2_Te_3_ nanowire	350 nm	10	<0.2 nA	~6–41 pA	--	49
individual ZnSe nanobelt	440 nm	30	~1.7 pA	<0.01 pA	--	50
thin film from Zn_2_SnO_4_ nanocubes	350 nm	5	43.2 nA	570 pA	76	This work

## References

[b1] Choi S.-H. *et al.* Amorphous zinc stannate (Zn_2_SnO_4_) nanofibers networks as photoelectrodes for organic dye-sensitized solar cells. Adv. Funct. Mater. 23, 3146–3155 (2013).

[b2] Rong A. *et al.* Hydrothermal synthesis of Zn_2_SnO_4_ as anode materials for Li-ion battery. J. Phys. Chem. B 110, 14754–14760 (2006). 16869583 10.1021/jp062875r

[b3] Zhao Y. *et al.* Preparation of hollow Zn_2_SnO_4_ boxes for advanced lithium-ion batteries. RSC Adv. 3, 14480–14485 (2013).

[b4] Cherian C. T., Zheng M. R., Reddy M. V., Chowdari B. V. R. & Sow C. H. Zn_2_SnO_4 _nanowires versus nanoplates: electrochemical performance and morphological evolution during Li-cycling. ACS Appl. Mater. Interfaces 5, 6054–6060 (2013). 23738585 10.1021/am400802j

[b5] Jiang Y. Q., He C. X., Sun R., Xie Z. X. & Zheng L. S. Synthesis of Zn_2_SnO_4_ nanoplate-built hierarchical cube-like structures with enhanced gas-sensing property. Mater. Chem. Phys. 136, 698–704 (2012).

[b6] Jiang Y. Q., Chen X. X., Sun R., Xiong Z. & Zheng L. S. Hydrothermal syntheses and gas sensing properties of cubic and quasi-cubic Zn_2_SnO_4_. Mater. Chem. Phys. 129, 53–61 (2011).

[b7] Shi L. & Dai Y. M. Synthesis and photocatalytic activity of Zn_2_SnO_4_ nanotube arrays. J. Mater. Chem. A 1, 12981–12986 (2013).

[b8] Zeng J. *et al.* Transformation process and photocatalytic activities of hydrothermally synthesized Zn_2_SnO_4 _nanocrystals. J. Phys. Chem. C 112, 4159–4167 (2008).

[b9] Xiang J., Lu J. W., Hu Y., Yan H. & Lieber C. M. Ge/Si nanowire heterostructures as high-performance field-effect transistors. Nature 441, 489–493 (2006). 16724062 10.1038/nature04796

[b10] Zhai T. Y. *et al.* A comprehensive review of one-dimensional metal-oxide nanostructure photodetectors. Sensors 9, 6504–6529 (2009). 22454597 10.3390/s90806504 PMC3312456

[b11] Konstantatos G. & Sargent E. H. Nanostructured materials for photon detection. Nat. Nanotechnol. 5, 391–400 (2010). 20473301 10.1038/nnano.2010.78

[b12] Averine S. V., Kumetzov P. I., Zhitov V. A. & Alkeev N. V. Solar-blind MSM-photodetectors based on Al_x_Ga_1−x_N/GaN heterostructures grown by MOCVD. Solid-State Electron. 52, 618–624 (2008).

[b13] Zeng X. *et al.* Low temperature synthesis of wurtzite zinc sulfide (ZnS) thin films by chemical spray pyrolysis. Phys. Chem. Chem. Phys. 15, 6763–6768 (2013). 23546181 10.1039/c3cp43470b

[b14] Wang X. *et al.* ZnO hollow spheres with double-yolk egg structure for high-performance photocatalysts and photodetectors. Adv. Mater. 24, 3421–3425 (2012). 22674659 10.1002/adma.201201139

[b15] Ghusoon M. A. & Parthasarathi C. Fabrication and characterization of thin film ZnO schottky contacts based UV photodetectors: a comparative study. J. Vac. Sci. Technol. B 30, 031206 (2012).

[b16] Chen K. J., Hung F. Y., Chang S. J. & Young S. J. Optoelectronic characteristics of UV photodetector based on ZnO nanowire thin films. J. Alloys Compd. 479, 674–677 (2009).

[b17] Xue H. L. *et al.* TiO_2_ based metal-semiconductor-metal ultraviolet photodetectors. Appl. Phys. Lett. 90, 201118 (2007).

[b18] Hu L. F. *et al.* Stacking-order-dependent optoelectronic properties of bilayer nanofilm photodetectors made from hollow ZnS and ZnO microspheres. Adv. Mater. 24, 5872–5877 (2012). 22933411 10.1002/adma.201202749

[b19] Chen M., Ye C. Y., Zhou S. X. & Wu L. M. Recent advances in applications and performance of inorganic hollow spheres in devices. Adv. Mater. 25, 5343–5351 (2013). 24089353 10.1002/adma.201301911

[b20] Chen H., Hu L. F., Fang X. S. & Wu L. M. General fabrication of monolayer SnO_2_ nanonets for high-performance ultraviolet photodetectors. Adv. Funct. Mater. 22, 1229–1235 (2012).

[b21] Hu L. F., Chen M., Fang X. S. & Wu L. M. Oil-water interfacial self-assembly: a novel strategy for nanofilm and nanodevice fabrication. Chem. Soc. Rev. 41, 1350–1362 (2012). 22076485 10.1039/c1cs15189d

[b22] Wang X., Tian W., Liao M. Y., Bando Y. & Golberg D. Recent advances in solution-processed inorganic nanofilm photodetectors. Chem. Soc. Rev. 43, 1400–1422 (2014). 24356373 10.1039/c3cs60348b

[b23] Zhang C. Y. *et al.* Facile one-step fabrication of ordered organic nanowire films. Adv. Mater. 21, 4172–4175 (2009).

[b24] Hu L. F., Ma R. Z., Ozawa T. C. & Sasaki T. Oriented monolayer film of Gd_2_O_3_:0.05 Eu crystallites: quasi-topotactic transformation of the hydroxide film and drastic enhancement of photoluminescence properties. Angew. Chem. Int. Ed. 48, 3846–3849 (2009). 10.1002/anie.200806206 19396854

[b25] Arumugam P. *et al.* Self-assembly and cross-linking of FePt nanoparticles at planar and colloidal liquid−liquid interfaces. J. Am. Chem. Soc. 130, 10046–10047 (2008). 18624408 10.1021/ja802178s

[b26] Xu L. *et al.* Hydrophobic coating- and surface active solvent-mediated self-assembly of charged gold and silver nanoparticles at water-air and water-oil interfaces. Phys. Chem. Chem. Phys. 11, 6490–6497 (2009). 19809681 10.1039/b820970g

[b27] Biswas S. & Drzal L. A novel approach to create a highly ordered monolayer film of graphene nanosheets at the liquid−liquid interface. Nano Lett. 9, 167–172 (2008). 19113892 10.1021/nl802724f

[b28] Zhang Y. J. *et al.* High performance ultraviolet photodetectors based on an individual Zn_2_SnO_4_ single crystalline nanowire. J. Mater. Chem. 20, 9858–9860 (2010).

[b29] Xu C. *et al.* Molecular synthesis of high-performance near-IR photodetectors with independently tunable structural and optical properties based on Si-Ge-Sn. J. Am. Chem. Soc. 134, 20756–20767 (2012). 23237361 10.1021/ja309894c

[b30] Yu Y. Q. *et al.* High-gain visible-blind UV photodetectors based on chlorine-doped n-type ZnS nanoribbons with tunable optoelectronic properties. J. Mater. Chem. 21, 12632–12638 (2011).

[b31] Kovalenko, Maksym V. *et al.* Colloidal HgTe nanocrystals with widely tunable narrow band gap energies: from telecommunications to molecular vibrations. J. Am. Chem. Soc. 128, 3516–3517 (2006). 16536514 10.1021/ja058440j

[b32] Dai P. C. *et al.* Band-gap tunable (Cu_2_Sn)*_x/3_*Zn*_1−x_*S nanoparticles for solar cells. Chem. Commun. 46, 5749–5751 (2010). 10.1039/c0cc00899k 20582379

[b33] Fan F. J. *et al.* Composition- and band-gap-tunable synthesis of wurtzite-derived Cu_2_ZnSn(S*_1−x_*Se*_x_*)_4_ nanocrystals: theoretical and experimental insights. ACS NANO 2, 1454–1463 (2013). 23350525 10.1021/nn3052296

[b34] Zhou H. L., Wu Y. J., Zhang W. & Wang J. Static hydrothermal crystallization of SUZ-4 zeolite in the presence of seed and tetraethylammonium hydroxide. Mater. Chem. Phys. 134, 651–656 (2012).

[b35] Alpuche-Aviles M. A. & Wu Y. Photoelectrochemical study of the band structure of Zn_2_SnO_4_ prepared by the hydrothermal method. J. Am. Chem. Soc. 131, 3216–3224 (2009). 19219993 10.1021/ja806719x

[b36] Hu Q. R. *et al.* Synthesis and photoluminescence of Zn_2_SnO_4_ nanowires. J. Alloys Compd. 484, 25–27 (2009).

[b37] Young D. L., Moutinho H., Yan Y. & Coutts T. J. Growth and characterization of radio frequency magnetron sputter-deposited zinc stannate, Zn_2_SnO_4_, thin films. J. Appl. Phys. 92, 310–319 (2002).

[b38] Fang X. S. *et al.* Single-crystalline ZnS nanobelts as ultraviolet-light sensors. Adv. Mater. 21, 2034–2039 (2009).

[b39] Liu B. D. *et al.* Bicrystalline ZnS microbelts. Cryst. Growth Des. 9, 2790–2793 (2009).

[b40] Ahn S. E. *et al.* Origin of the slow photoresponse in an individual sol-gel synthesized ZnO nanowire. Appl. Phys. Lett. 90, 153106 (2007).

[b41] Ahn S. E. *et al.* Photoresponse of sol-gel-synthesized ZnO nanorods. Appl. Phys. Lett. 84, 5022–5024 (2004).

[b42] Wang W. S., Wu T. T., Chou T. H. & Chen Y. Y. A ZnO nanorod-based SAW oscillator system for ultraviolet detection. Nanotechnology 19, 135503 (2009). 19420502 10.1088/0957-4484/20/13/135503

[b43] Hu L. F., Wu L. M., Liao M. Y. & Fang X. S. ZnS nanostructure arrays: a developing material star. Adv. Mater. 23, 1988–1992 (2011). 21274908 10.1002/adma.201003624

[b44] Wang J., Wang Y., Cao F., Guo Y. & Wan L. Synthesis of monodispersed wurtzite structure CuInSe_2_ nanocrystals and their application in high-performance organic−inorganic hybrid photodetectors. J. Am. Chem. Soc. 132, 12218–12221 (2010). 20712316 10.1021/ja1057955

[b45] Fang X. S., Bando Y., Gautam U. K., Ye C. H. & Golberg D. Inorganic semiconductor nanostructures and their field-emission applications. J. Mater. Chem. 18, 509–522 (2008).

[b46] Huang Y., Duan X. F. & Lieber C. M. Nanowires for integrated multicolor nanophotonics. Small 1, 142–147 (2005). 17193365 10.1002/smll.200400030

[b47] Kind H., Yan H., Messer B., Law M. & Yang P. Nanowire ultraviolet photodetectors and optical switches. Adv. Mater. 14, 158–160 (2002).

[b48] Fang X. S. *et al.* New ultraviolet photodetector based on individual Nb_2_O_5_ nanobelts. Adv. Funct. Mater. 21, 3907–3915 (2011).

[b49] Wang Z. X., Safdar M., Jiang C. & He J. High-performance UV-visible-NIR broad spectral photodetectors based on one-dimensional In_2_Te_3_ nanostructures. Nano Lett. 12, 4715–4721 (2012). 22908854 10.1021/nl302142g

[b50] Fang X. S. *et al.* High-performance blue/ultraviolet-light-sensitive ZnSe-nanobelt photodetectors. Adv. Mater. 21, 5016–5021 (2009). 10.1002/adma.200902126 25376153

