# Supplementary material for: Band Gap Tunable Zn2SnO4 Nanocubes through Thermal Effect and Their Outstanding Ultraviolet Light Photoresponse
Source: Sci Rep. 2014 Oct 30;4:6847. doi: 10.1038/srep06847 (PMC4213775; doi:10.1038/srep06847)
Supplement: Supplementary Information — files [file srep06847-s1.doc]

Supporting Information

Band Gap Tunable Zn_2_SnO_4_ Nanocubes through Thermal Effect and Their Outstanding Ultraviolet Light Photoresponse

Yan Zhao^1a^, Linfeng Hu^1a^, Hui Liu^1^, Meiyong Liao^2^, Xiaosheng Fang^1^ & Limin Wu^1*^

^1^ Department of Materials Science, Advanced Materials Laboratory, Fudan University, Shanghai 200433, P. R. China, E-mail: [lmw@fudan.edu.cn](mailto:lmw@fudan.edu.cn)

^2^ Optical and Electronic Materials Unit, National Institute for Materials Science (NIMS), Namiki 1-1, Tsukuba, Ibaraki 305-0044, Japan

Methods

**Materials:** Zinc acetate (Zn(CH_3_COO)_2_•2H_2_O, 90 %), stannic chloride (SnCl_4_•5H_2_O, 99 %), sodium dodecyl benzene sulfonate (SDBS, C_18_H_29_NaO_3_S) and tetraethylammonium hydroxide (C_8_H_20_N•OH, 25 % in water) were purchased from commercial suppliers (Sinopharm Chemical Regent Co., Ltd. and Aladdin Industrial Reagent Corporation) and used as received without further puriﬁcation.

**Synthesis of Zn_2_SnO_4_ nanocubes:** Zn_2_SnO_4_ nanocubes were synthesized by a hydrothermal method with some changes of experimental conditions.^5^ Brieﬂy, 1 mmol of Zinc acetate, 0.5 mmol of stannic chloride and 1 mmol of SDBS were added into a mixed solution of ethanol (15 mL) and distilled water (15 mL) in a conical flask and put it in a water bath under magnetic stirring at 60 °C. The mixture was stirred for 30 min with a pH of 2.95. Then, 3.0 mL of TEAH was added dropwise to the stirred solution and the pH turned to 13.3. After continuously stirred for 1 h, the suspension was then transferred into a 50 mL Teﬂon-lined stainless steel autoclave, sealed tightly, and maintained at 220 °C for 5 h. After the autoclave was cooled to room temperature naturally, the resultant precipitates were collected by centrifuging at 3000 rpm for 10 min and washed more than 6 times with ethanol and distilled water in an ultrasonic cleaning bath for 5 min, respectively. Then, it was dispersed in 10 mL of ethanol for further characterization. Other Zn_2_SnO_4_ crystals, such as nanoparticles, nanoplates and large size of nanocubes, were prepared in a similar way except for the various reaction conditions. The yield of the product was calculated by following equation:

$$Y=\frac{m}{M}\times100$$

Where *Y* represents the yield (%), *m* is the actual molar weight (mmol) of the obtained product, and *M* is the molar weight (mmol) of the Sn^2+^, which is equal to the theoretical molar weight of Zn_2_SnO_4_ nanocubes.

**Preparation of Zn_2_SnO_4_ nanocube-based nanofilms:** Zn_2_SnO_4_ nanocube-based nanofilm was fabricated using an oil–water interfacial self-assembly method. Typically, a certain amount of Zn_2_SnO_4_ nanocubes (dispersed in ethanol) was transferred into a 1 mL injection syringe. Then, 40 mL water and 5 mL of hexane were added into a 50 mL glass, respectively, to produce a hexane-water interface. After that, the Zn_2_SnO_4_ nanocubes were added to the interface at a very slow rate by the 1 mL syringe. The Zn_2_SnO_4_ nanocubes were gradually trapped at the interface to form closely-packed nanofilm. After most of hexane was removed, the assembled nanofilm was kept at the interface for another 10 min and transferred onto different substrates by a lifting method. Four as-prepared Zn_2_SnO_4_ nanofilms were obtained by this method. Then, these films were allowed to dry overnight and calcined at 60 °C, 200 °C, 300 °C and 500 °C for 1 h (1 °C min^−1^ and max cooling ramp), respectively.

**Construction of Zn_2_SnO_4_ nanocube-based nanofilm photodetectors:** The as-transformed Zn_2_SnO_4_ nanofilms were transported to an electric gun deposition system (ULVAC UEP-3000-2C). The Ti/Au (100 nm/100 nm) microelectrodes (distance ≈ 30 μm) were patterned on the top of each Zn_2_SnO_4_ nanofilm using optical lithography with the assistance of a pre-designed mask and electron-beam deposition followed by a lift-off process. The procedures are schematically illustrated in Fig. 1. The current density-voltage (*J-V*) characteristics of the Zn_2_SnO_4_ nanofilm photodetector were measured using an Advantest picoammeter R8340A and a dc voltage source R6144. The spectral responses for different wavelengths were recorded by using a xenon lamp (500 W). The time-dependent photoresponses of the device were measured using a current meter after shutting off the UV light. The incident light power was calibrated using an UV enhanced Si photodiode.

**Characterization:** The morphologies of the products were taken by using field-emission scanning microscope (FESEM, JEOL JSM-6701F) and transmission electron microscope (TEM, JEOL JEM-2100F) equipped with an X-ray energy dispersive spectrometer (EDS). The phase of the sample was determined using a Rigaku D/max-rB X-ray diffractometer using Cu Kα radiation (*λ* = 0.15406 nm). The UV−vis adsorption spectra were obtained using Hitachi U-4100 spectrophotometry. The Zn/Sn ratio in Zn_2_SnO_4_ was determined by ICP-OES; Zn_2_SnO_4_ samples were digested in 3:1 v/v HCl:HNO_3_ (conc) and ultimately diluted to ca. 400 ppb in 2 % HNO_3_ v/v.


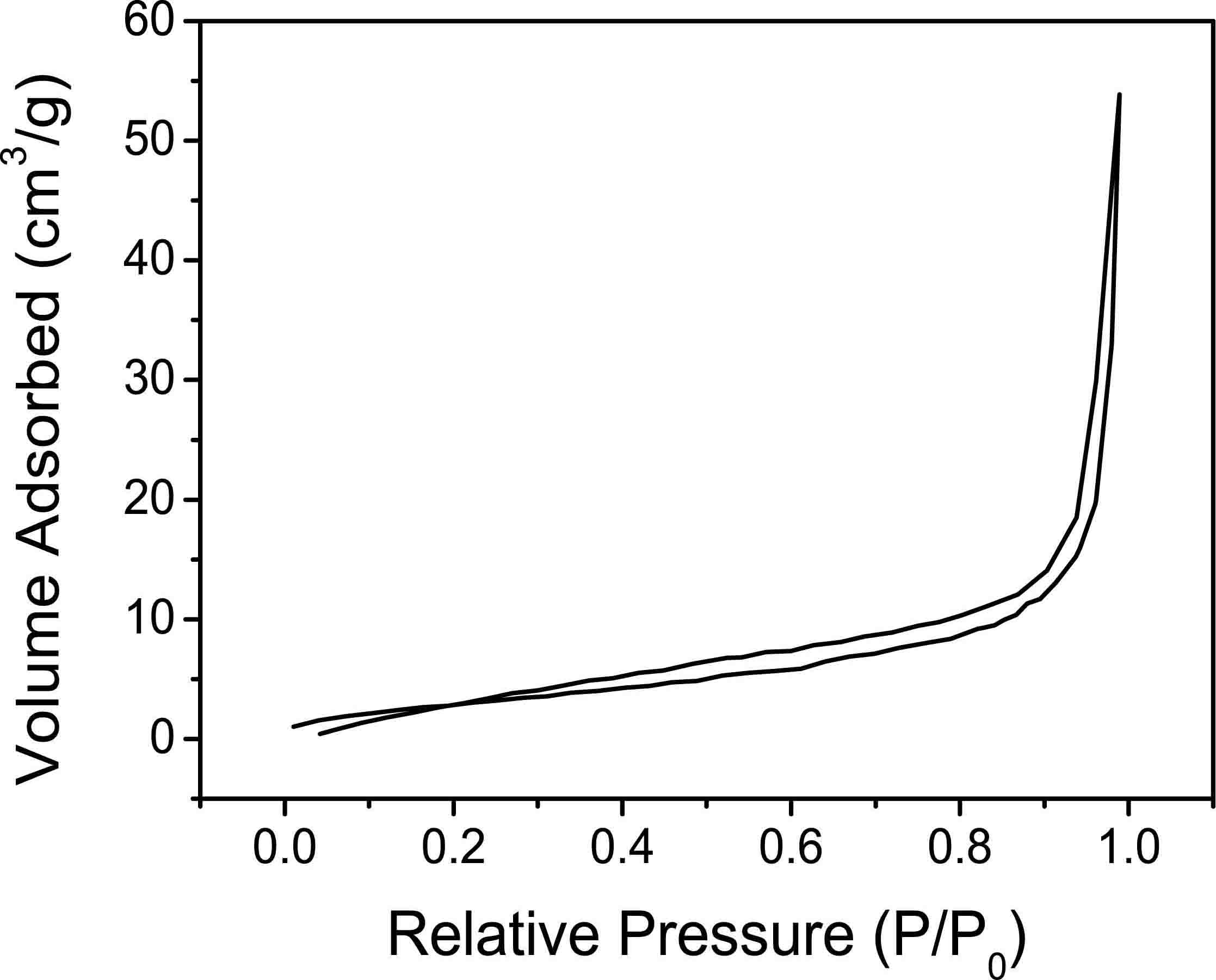


**Figure S1:** N_2_ adsorption–desorption isotherms of Zn_2_SnO_4_ nanocubes.


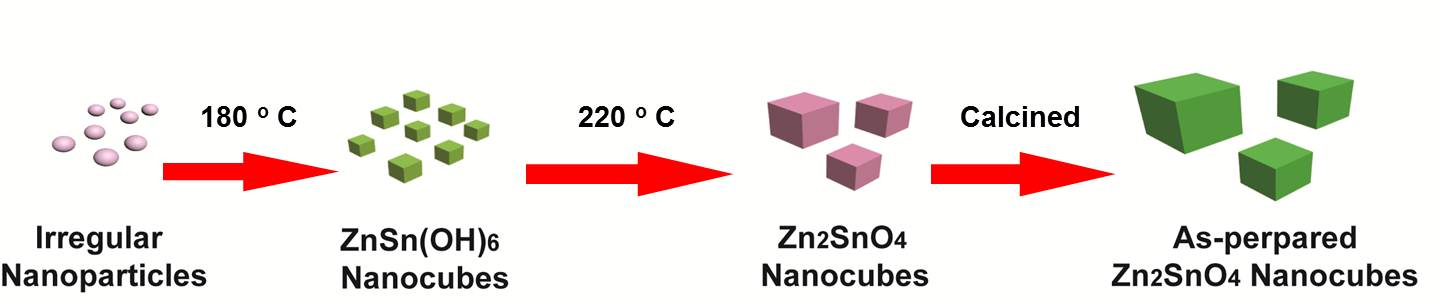


**Figure S2:** Schematic illustration for the possible formation mechanism of the as-prepared Zn_2_SnO_4_ nanocubes.


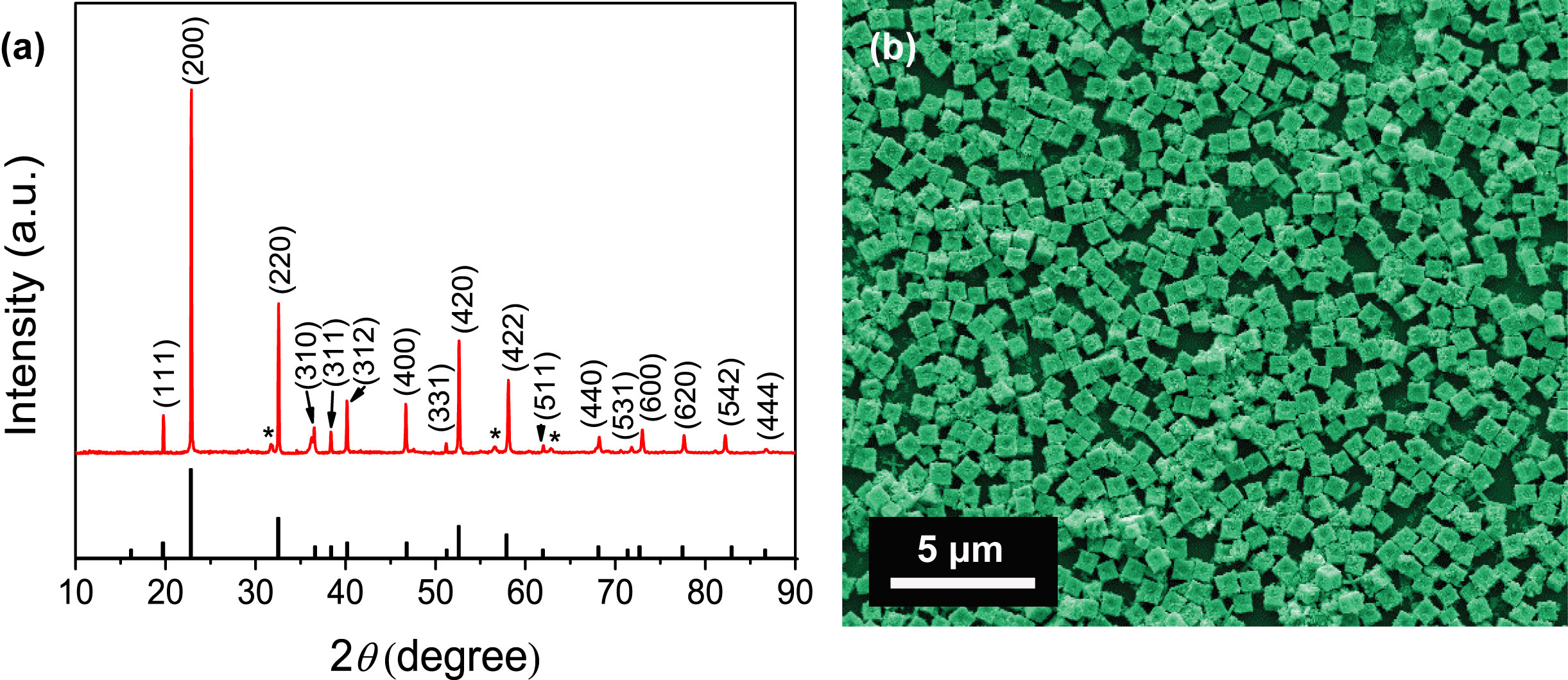


Figure S3: (a) The XRD pattern and (b) Related SEM image of the ZnSn(OH)_6_ nanocubes. JCPDS 33-1376 pattern is shown for comparison (vertical lines). * denotes diffraction peak of hexagonal ZnO.


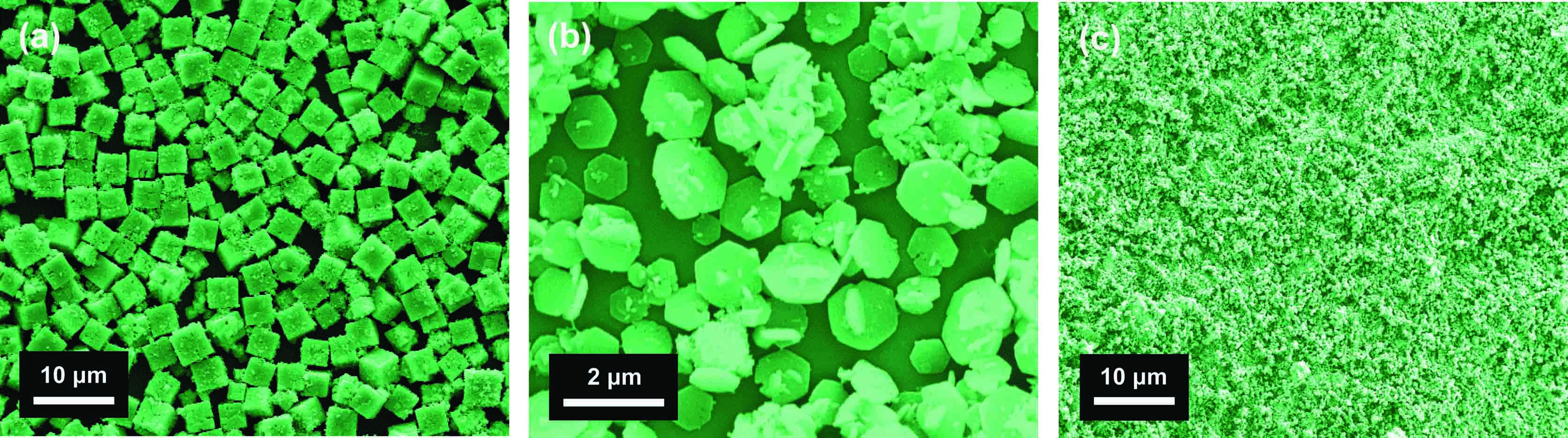


**Figure S4:** The SEM images of the monolayer Zn_2_SnO_4_ nanostructure-based films prepared at: (a) 220 ^o^C for 8 h; (b) 200 ^o^C for 20 h, without SDBS; (c) 220 ^o^C for 5h, without SDBS.


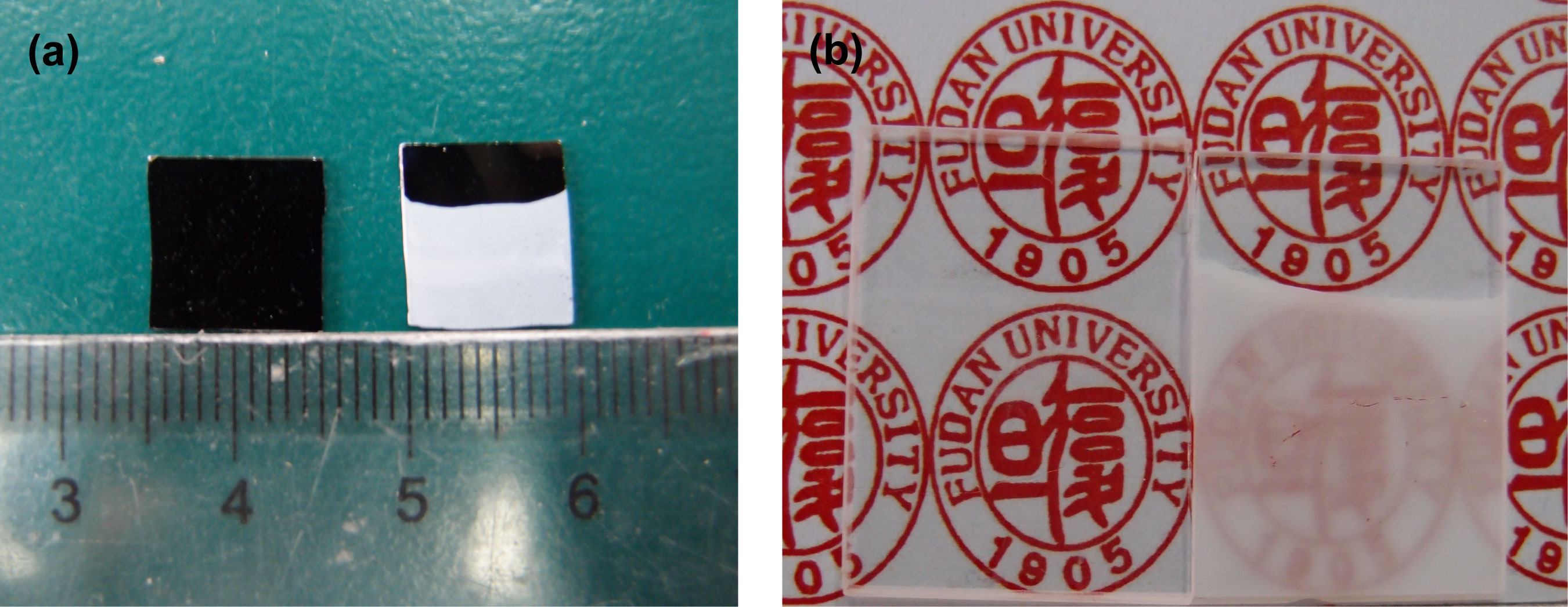


**Figure S5:** The optical microscopy images of the Zn_2_SnO_4_ monolayer nanofilm deposited on **(a)** silicon substrates and **(b)** quartz substrates under natural light, respectively.
